# Zirconia-reinforced glass ionomer restorations in molar incisor hypomineralization: a randomized controlled clinical trial

**DOI:** 10.1007/s00784-025-06352-y

**Published:** 2025-05-08

**Authors:** Reham A. Mahfouz, Azza G. Hanno, Amina M. Abd El Rahman

**Affiliations:** Department of Pediatric Dentistry, Faculty of Dentistry, Alexandria University, El Khartoum Square, Alexandria, 21131 Egypt

**Keywords:** Zirconia-reinforced glass ionomer cement, Equia forte, Molar incisor hypomineralization, Atraumatic restorative treatment, Glass hybrid restorations, Remineralization, Hypersensitivity

## Abstract

**Objectives:**

This study aimed to evaluate the effectiveness of zirconia-reinforced glass ionomer (ZrGI), in reducing pain and sensitivity, and to assess its clinical success in comparison to glass-hybrid glass ionomer (GhGI) in hypomineralized permanent first molars (HPFM) following the Atraumatic Restorative Treatment (ART) approach.

**Materials and Methods:**

This randomized controlled clinical trial included 7–10-year-old children. Eighty-eight HPFMs with evidence of caries, with or without hypersensitivity were included. These molars scored (2a), (2b), (4a), or (4b) according to the MIH Treatment Need Index (MIH-TNI). Selective caries removal was performed using the ART protocol. The test group received ZrGI (Zirconomer®) restorations, and the control group received GhGI (Equia Forte®) restorations. Pain and sensitivity were assessed pre-operatively, using the Visual Analogue Scale (VAS), then re-assessed post-operatively after 1 week and after 3,6,9, and 12 months (M). Clinical success was evaluated, starting from the 3-month visit, using the ART evaluation criteria.

**Results:**

After 12M, the median pain scores for the ZrGI gp., (Q1-Q3; 0.00 – 0.00) and GhGI gp., (Q1-Q3; 0.00 – 0.00) showed no statistically significant difference, (*p* = 0.329). Median sensitivity scores for the ZrGI gp., (Q1-Q3; 0.00 – 5.00) and the GhGI gp., (Q1-Q3;0.00 – 3.50), also showed no statistically significant difference, (*p* = 0.344). No significant difference was found between the 12M success rate of ZrGI (86.4%) and GhGI (84.1%), (*p* = 0.765).

**Conclusion:**

Regarding the reduction of pain and sensitivity, and clinical success, zirconia-reinforced GIC was as effective as glass hybrid GIC in restoring HPFM, using the ART approach.

**Clinical significance:**

Zirconia-reinforced glass ionomer cement may be a promising restorative material due to its favorable physical properties and acceptable clinical results, as shown in this study. It may be used as an interim restoration in hypomineralized permanent first molars and holds potential for even wider applications in clinical pediatric dentistry.

**Trial Registration:**

Please refer to this study by its ClinicalTrials.gov identifier: NCT05494749

**Supplementary Information:**

The online version contains supplementary material available at 10.1007/s00784-025-06352-y.

## Introduction

Molar incisor hypomineralization (MIH) is a qualitative enamel defect that results from alterations during amelogenesis [1]. It affects one or more of the permanent first molars (PFMs) and may involve the permanent incisors [2]. Diagnosis of MIH is confirmed by the presence of characteristic features, such as demarcated yellowish-white opacities or post-eruptive enamel breakdown (PEB), in at least one PFM [2]. The global prevalence of MIH is estimated to be 12.9% (11.7–14.3%) [3]. Researchers reported that dental hypersensitivity and pain have a negative impact on oral health-related quality of life in MIH-affected children [4].

The European Academy of Pediatric Dentistry (EAPD) released an updated policy document on the best management strategies for MIH [5] recommending resin-based fissure sealants as a first-line approach to prevent dental caries and PEB [6, 7]. Composite restorations, stainless steel metal crowns, and onlays may be used to restore carious and hypomineralized areas in MIH-affected teeth. Scheduled extractions may be a last resort in severely affected non-restorable molars.

Contemporary dental practice is directed towards the application of minimally invasive techniques [5]. Atraumatic Restorative Treatment (ART) reflects a shift from the traditional drill and fill strategy [8]. This approach has shown promising results using high-viscosity or glass-hybrid glass ionomer cement (GIC) [9, 10]. HPFMs may benefit from the removal of the carious/hypomineralized enamel and decomposed dentine using this simple, time-efficient approach [8]. In underprivileged areas that do not have access to routine dental care, ART is convenient for caries removal in MIH-affected children since only hand instruments are required [11].

GICs may be used as interim restorations in hypomineralized permanent first molars (HPFM) [5, 12]. GIC is well known for its numerous merits like chemical adhesion to enamel and dentine, and enhancing remineralization through fluoride release [13–15]. Difficult dental situations where ideal isolation cannot be achieved seem to provide an advantage with GIC restorations, being a moisture-tolerant restorative material [16]. Another advantage is the ease of placing GICs in partially erupted first permanent molars [17].

The fact that GIC restorations are not recommended in stress-bearing areas, limits their use in MIH-affected molars [18]. There is a high demand for an enforced type of GIC, capable of withstanding the increasing occlusal loads that MIH-affected molars are exposed to in the early-to-late mixed dentition stage. The success of glass hybrid glass ionomer (GhGI) restorations in HPFMs was investigated in several clinical trials, where success rates of 98.3%, and 77.4% were reported [10, 19]. Zirconia-reinforced glass ionomer (Zirconomer Improved® and Zirconomer P®) [20, 21] is a recent modification of GICs containing zirconia particles for enhancement of its mechanical properties [22, 23]. Arefein et al. [24] reported that ZrGI restorations may be the best available alternative to GICs, describing it as the ‘white amalgam’. Bhatia et al. [25] reported in their in-vitro study that ZrGI is a promising restoration in stress-bearing areas. The ease of application of ZrGI, its chemical setting nature, and its claimed promising physical properties advocate its use with the ART model. Studies reporting on the clinical success of ZrGI are scarce; this fueled our endeavors to address this knowledge gap. To date, our research is the first to highlight the success rate of ZrGI restorations in the context of hypomineralized molars. The present study aims to evaluate the effectiveness of ZrGI in reducing pain and sensitivity (primary outcome), and to assess its clinical success (secondary outcome) in comparison to GhGI restorations in MIH-affected molars using the ART approach. The Null hypothesis states that no difference exists between ZrGI and GhGI regarding the reduction of pain and sensitivity, and clinical success in HPFMs, after a 12-month follow-up period.

## Materials and methods

Ethical approval was obtained from the Research Ethics Committee of Alexandria University, Faculty of Dentistry (IRB No. 00010556-IORG 0008839). This clinical study abides by the Declaration of Helsinki. The research protocol and any possible complications were explained to the participants and their parents. A written informed consent [26] was obtained from all parents/legal guardians of children before the study commenced.

**Fig. 1:**
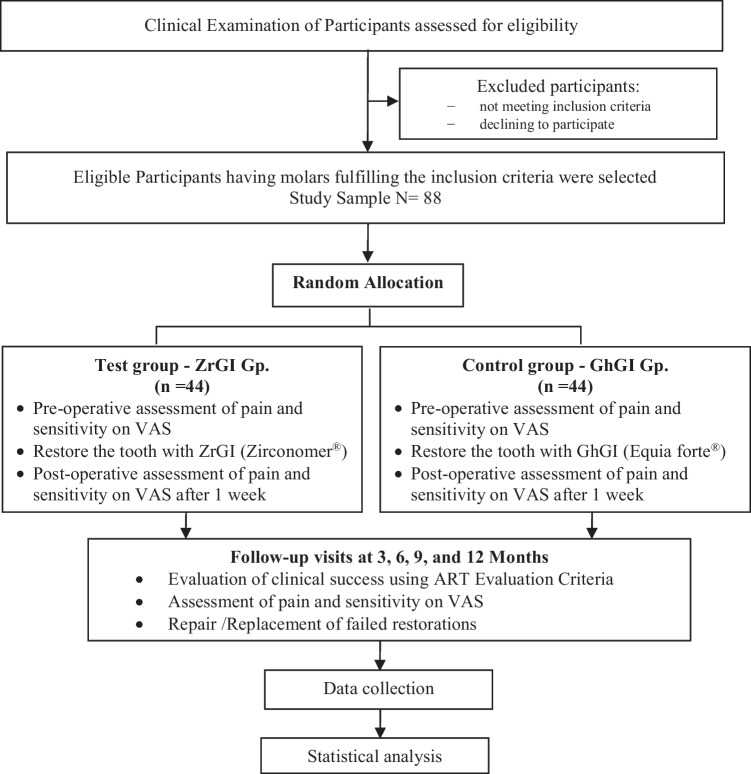
Study Flow Diagram

The study was designed to be a two-arm randomized controlled clinical trial, with a 1:1 allocation ratio. It was set up and reported according to the CONSORT [27] guidelines and the CONSORT-PRO 2022 [28] extension for patient-reported outcomes. Participants were recruited from the Pediatric Dentistry and Dental Public Health Department, Faculty of Dentistry, Alexandria University, Egypt. Figure 1 shows the clinical trial flow diagram [27].

### Sample size estimation

The sample size was estimated based on Rosner’s method [29] according to Grossi et al. [10], and Elsawy et al. [30], assuming a 5% alpha error and 80% study power. The initial sample size for each group was 35 molars adding up to 70 molars as a total sample size. To make up for lost-to-follow-up cases, or drop-outs, the total sample size was raised to 88 molars.

### Inclusion criteria

Children 7–10 years old diagnosed with MIH according to the EAPD evaluation criteria [31] were included in this study. Children were free from any systemic disease (ASA I) [32], and showed positive or definitely positive behavior according to the Frankl rating scale [33]. Eighty-eight (88) HPFMs were included in this study; only one maxillary or mandibular HPFM was selected per patient. The molars scored (2a), (2b), (4a), or (4b) according to the MIH Treatment Need Index (MIH-TNI) [34]-*refer to Supplementary Information (SI).* These scores indicate that the enamel defect was less than one-third of the HPFM or between one-third to two-thirds of the molar. The defects presented with or without hypersensitivity. Carious molars scoring 5 or 6 on the ICDAS II [35, 36], with no clinical or radiographic evidence of pulpal involvement, were included. Patients who presented with non-restorable permanent first molars or those indicated for extraction were excluded.

### Randomization, concealment, and blinding

Permuted block randomization [37] was performed using Research Randomizer online software [38]. Patients were randomly and equally allocated to groups A or B with a (1:1 ratio). Blocks of multiple sizes, sizes 4 and 6, were used to decrease predictability.

Each child was given a serial number used in the allocation. These numbers were written on identical sheets of paper denoting the group to which each child was allocated and placed inside opaque envelopes carrying the respective names of the children. A trial-independent personnel was assigned to keep the envelopes and unfold them only at the time of the treatment session so that the group, to which the child was allocated was concealed from the operator.

In group A, the Test group, molars were assigned to receive ZrGI restorations. In group B, the Control group, the teeth were assigned to receive GhGI restorations.

This study was triple-blinded. The assessors, the patient, and the statistician were blinded to the treatment groups. Similar shades of both restorative materials were chosen to ensure blinding of the assessors.

### Training and calibration

The operator (R.M.), who is the primary investigator in this study, was trained and calibrated regarding the diagnosis of MIH according to the EAPD criteria [31]. The operator was also trained and calibrated regarding assessment of the severity of molar hypomineralization according to the MIH-TNI [34]. Kappa test result for Intra-examiner reliability regarding MIH-TNI scores was (0.82). Calibration was carried out by examining 10 molars in two successive settings, these were not included in the study sample.

Two experienced pediatric dentists, A.H. and A.A. (the study supervisors), were assigned to assess the restorations according to the ART evaluation criteria, *(see SI)*. Kappa test results for intra-examiner reliability of each assessor were (0.75) and (0.87) [39]. The kappa-statistic value for inter-examiner agreement was (0.81) indicating good agreement.

### Pre-operative assessment

Using the Visual Analogue Scale (VAS) [40] children rated the subjective perception of pain from 0 to 10, where 0 = no pain, and 10 = unbearable pain. The pre-operative assessment of sensitivity was performed using the air blast test [41], which was performed using the dental air syringe at 30 psi pressure and 23ºc ± 3ºc temperature for 1 s. The syringe was kept 1 cm away from the tooth. The child was asked to rate his experience using the VAS, the child’s response was recorded as a score from 0 to 10.

### Operative procedure

Moisture control was achieved using cotton rolls, and suction tips. Carious tissue involving hypomineralized enamel, was removed with sharp excavators of 1 mm in diameter. Any residual hypomineralized or unsupported enamel was removed with a hatchet specifically developed for the ART approach. The restoration margin was placed in sound enamel.

The caries removal process followed the principles of Minimum Intervention Dentistry, where the tissue was removed selectively, depending on the cavity depth [42]. Removal of hypomineralized, unsupported, carious enamel and dentine was performed by hand instruments such as sharp spoon excavators, chisels, and hatchets [8]. An alternative method for caries removal was the modified ART(mART) [43] approach in which 0.5 round diamond or carbide burs mounted on a low-speed rotary handpiece were used. When necessary, anesthesia was administered.

Molars allocated to the test gp. received ZrGI restorations (Zirconomer® Improved, Shofu Inc., Tokyo, Japan). The filling material was prepared and mixed according to the manufacturer’s instructions. After the cavity was filled, the restoration surface was coated with cocoa butter for protection against moisture. Polishing with abrasive discs and stones was done in subsequent visits.

Molars allocated to the control gp. received GhGI restorations (EQUIA Forte®, GC, Tokyo, Japan). Capsules were mixed in a capsule mixer at 4000 rpm for 10 s. Its content was then inserted into the cavity according to the manufacturer’s instructions. After complete setting of the material, a coat was applied (EQUIA Forte® Coat,GC America, USA), then light-cured for 20 s.

### Evaluation

After 1 week, assessment of pain and sensitivity was performed. Subsequent recall visits were scheduled, and the assessments of pain and sensitivity using VAS were repeated and recorded at the 3-, 6-, 9-, and 12-month recall visits.

Starting from the 3-month recall visit, restorations were inspected, by the two assessors, and given scores from 0 to 5 using the ART evaluation criteria [44]. The depth of marginal defects was measured using the 0.5 mm ball-end of a metal community periodontal index (CPI) probe.

Restorations scoring 0 or 1 were considered successful, other scores ≥ 2 were considered as failed. In case of disagreement between the assessors, the restoration was re-examined until a consensus was reached. Differences were predominantly concerning scores 0 and 1 of the ART criteria. In subsequent follow-up visits, any faulty restoration was replaced, and the molar was excluded from the study.

### Statistical analysis

Normality of data was checked using *Shapiro Wilk test* and Q-Q plots. Age was normally distributed; therefore, it was presented using mean and standard deviation. VAS scores were not normally distributed; therefore, data summarization included the median, minimum and maximum, as well as the first and third quartiles. Gender, arch type, and ART success/failure rate were presented using frequency and percentage. Independent t-test was used to analyze age. Comparison between groups regarding VAS scores was done using *Mann Whitney U test,* whereas changes across follow-up time points were assessed using *Friedman test,* followed by pairwise comparisons using *Bonferroni correction*. Differences in gender, arch type, and ART success/failure rates between groups were analyzed using *Pearson Chi-Square test* and *Fisher’s Exact test*. *Cochran’s Q test* was employed to analyze differences in ART success/failure rates within groups. The overall ART survival between groups was evaluated using *Kaplan–Meier survival analysis* and *Log-rank test*. Examiners’ reliability was tested using *Kappa statistic test*. All tests were two-tailed, and the significance level was set at *p-*value < 0.05. Data were analyzed using IBM SPSS version 23, Armonk, NY, USA, and MedCalc for Windows, version 19.4 (MedCalc Software, Ostend, Belgium).

## Results

Eighty-eight molars were included in this study. No sample attrition was recorded throughout the entire study period. The mean age for the test and control groups was (7.88 ± 0.73) and (7.85 ± 1.00) respectively. Age, gender, and molar position in the arch were normally distributed, *(see SI)*.

Table 1 shows that the median VAS pain scores in both test and control groups decreased at the end of the follow-up period. Among-group comparisons of pain scores at baseline and in each follow-up visit, revealed no significant differences except for the 1 st-week visit, in which the ZrGI gp. recorded higher pain scores, (*p* = 0.022). Pairwise comparisons of pain scores in both groups showed significant differences between baseline values and those at each recall visit, (*p* < 0.0001), *(see SI)*.

**Table 1:** Pain scores (top), and sensitivity scores (bottom) in ZrGI and GhGI groups throughout the study period

Time Intervals	ZrGI (Test) (*n* = 44)		GhGI (Control) (*n* = 44)		*p-valueǂ*
	Median (Min – Max)	Q1 – Q3	Median (Min – Max)	Q1 – Q3	
Baseline	4.00 (0.00–10.00)	0.00–8.00	4.00 (0.00–10.00)	0.00–8.00	0.541
	3.00 (0.00–10.00)	0.00–7.00	4.00 (0.00 − 10.00)	0.00–6.00	0.655
1 Week	0.00 (0.00–6.00)	0.00–0.00	0.00 (0.00–0.00)	0.00–0.00	0.022*
	0.00 (0.00—6.00)	0.00–0.00	0.00 (0.00–10.00)	0.00–0.00	0.155
3 Months	0.00 (0.00–4.00)	0.00–0.00	0.00 (0.00–6.00)	0.00–0.00	0.963
	0.00 (0.00–6.00)	0.00–0.00	0.00 (0.00–6.00)	0.00–0.00	0.482
6 Months	0.00 (0.00–4.00)	0.00–0.00	0.00 (0.00–6.00)	0.00–0.00	0.577
	0.00 (0.00–6.00)	0.00–2.00	0.00 (0.00–4.00)	0.00–0.00	0.046*
9 Months	0.00 (0.00–4.00)	0.00–0.00	0.00 (0.00–10.00)	0.00–0.00	0.411
	0.00 (0.00–10.00)	0.00–4.00	0.00 (0.00–8.00)	0.00–0.00	0.152
12 Months	0.00 (0.00–8.00)	0.00–0.00	0.00 (0.00–10.00)	0.00–0.00	0.329
	0.00 (0.00–10.00)	0.00–5.00	0.00 (0.00–8.00)	0.00–3.50	0.344
*Pain p-*value^§^	< 0.0001*		< 0.0001*		
*Sensitivity* *p-*value^§^	< 0.0001*		< 0.0001*		

*Statistically significant difference at *p*-value < 0.05, ǂMann Whitney U test, §Friedman Test

Table 1 indicates a significant decrease in sensitivity scores in both groups after the 1 st-week recall visit. There was no statistically significant difference between the groups except at the 6-month recall visit, where sensitivity scores reported in the ZrGI gp., (Q1-Q3: 0.00–2.00) were significantly higher than the GhGI gp., (Q1-Q3: 0.00–0.00), (*p* = 0.046).

Pairwise comparisons within groups revealed that sensitivity scores in both groups were significantly lower than their baseline values except for the 9 and 12M visits in the ZrGI gp., where no significant differences were recorded, *(see SI)*.

**Fig. 2:**
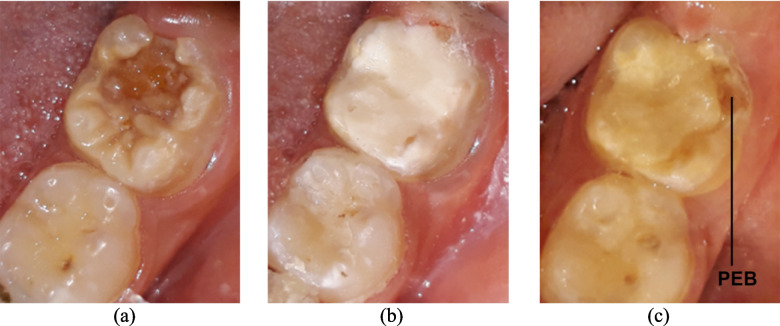
**(a)** carious hypomineralized permanent first molar, (**b**) after restoration with ZrGI, and (**c**) after 12 M; notice the integrity of the restoration with evidence of post-eruptive enamel breakdown (PEB) at the distobuccal cavity margins

**Fig. 3:**
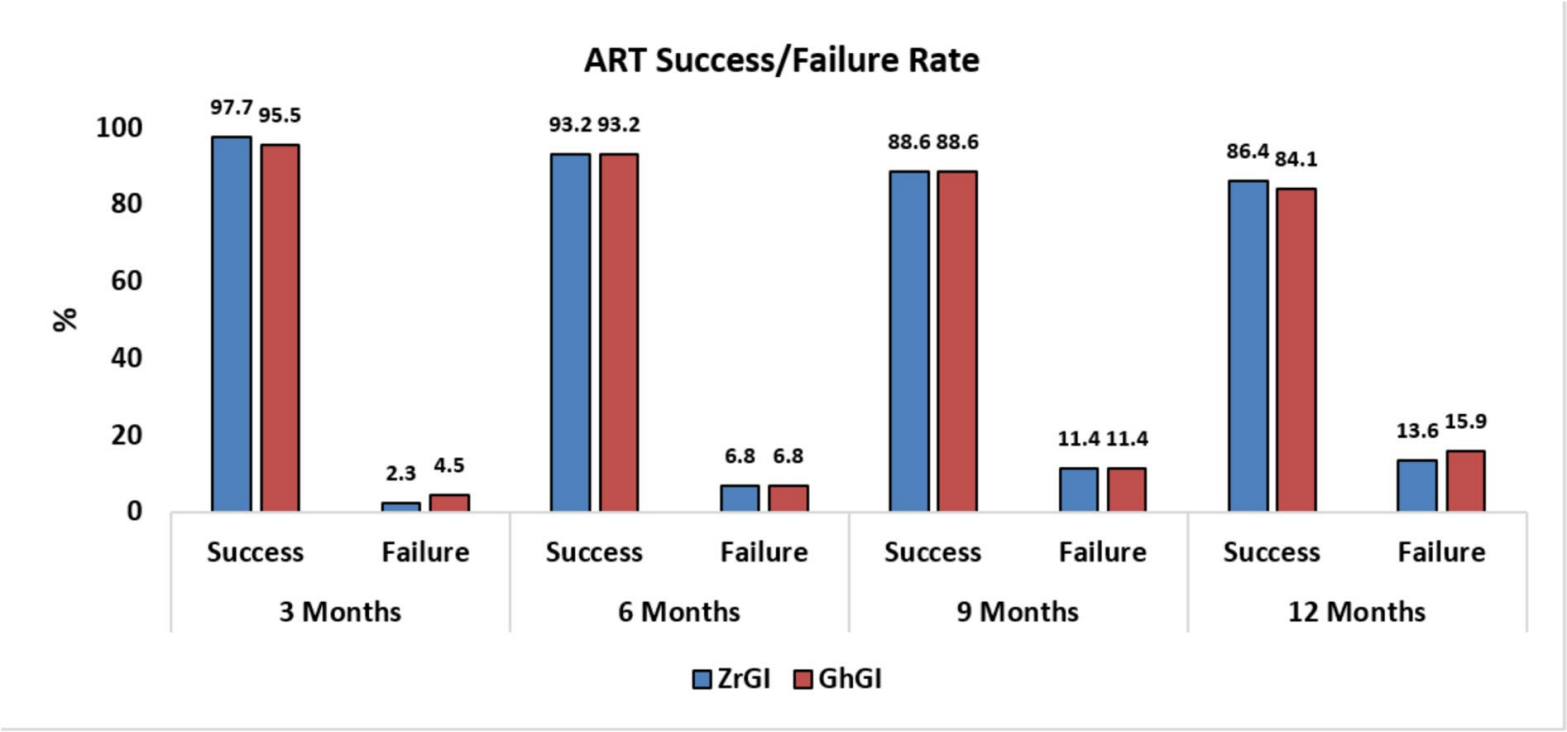
Percentage of successful/failed ART restorations in both groups

Figure 2 shows photographs of a hypomineralized molar restored with ZrGI pre-operative, immediately after restoration, and after 12 months, *(additional photos showing both groups are provided in SI)*. At the end of this study, 6 restorations in the ZrGI gp. and 7 in the GhGI gp. were considered as failed, according to the ART evaluation criteria. The success rate after 12M for ZrGI was 86.4% compared to 84.1% success rate in the GhGI group, Fig. 3. Results of Fisher’s Exact test, and Pearson Chi-Square test show that no significant differences were found between groups, *(see SI)*. Pairwise comparisons within each group regarding the ART scores show that the difference in success rates between the 3- and 12-month visits was statistically significant in both groups, *(see SI)*.

**Fig. 4:**
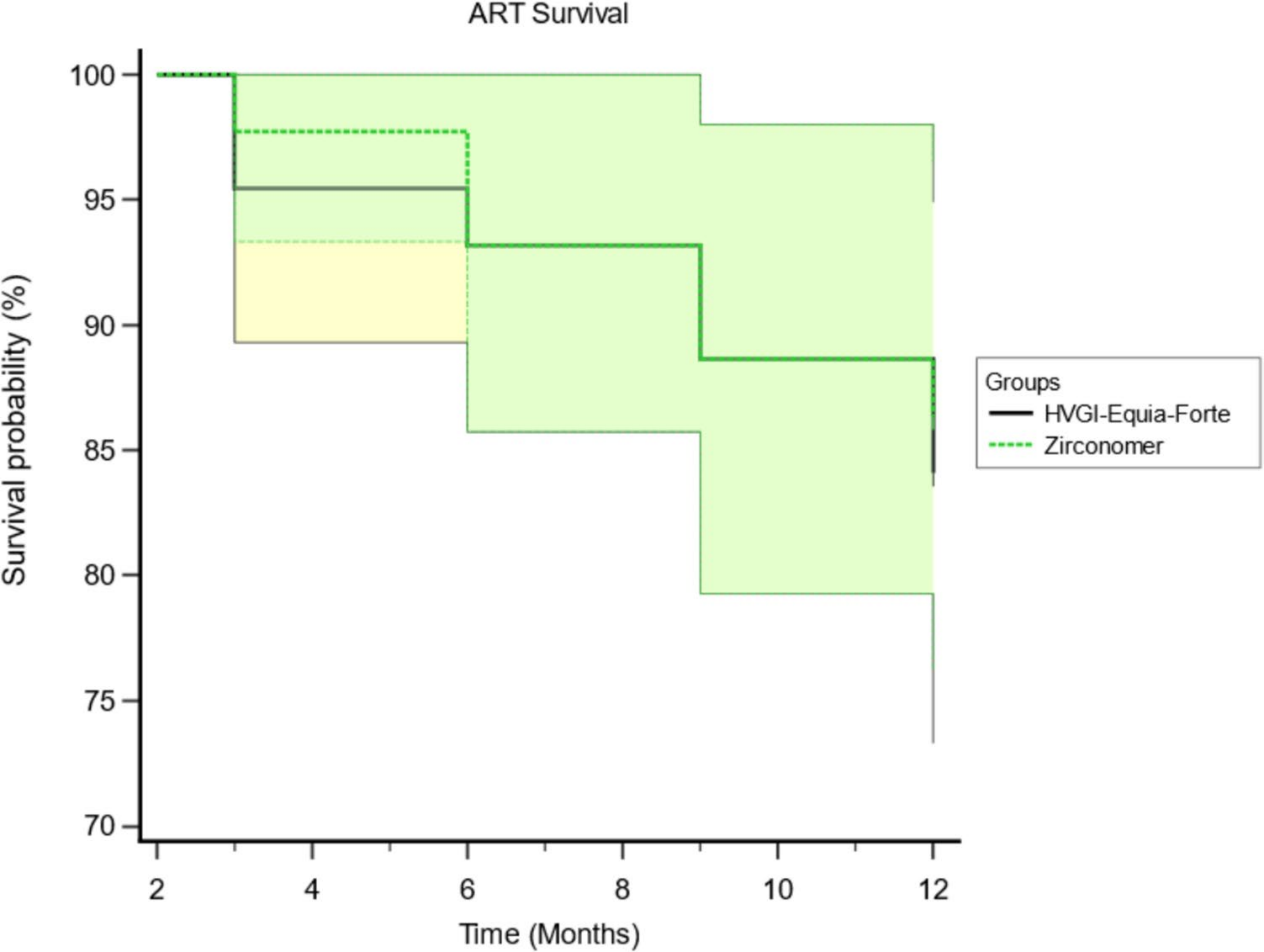
Survival analysis of ZrGI and GhGI restorations according to code 2 in the ART evaluation criteria

A Kaplan–Meier survival analysis for the 12M follow-up of ZrGI and GhGI restorations is shown in Fig. 4. The event assigned for this analysis was detection of a marginal defect more than 0.5 mm in depth (code 2 in the ART evaluation criteria) indicating restoration failure. The mean survival times of ZrGI and GhGI restorations were 11.39 and 11.32 months respectively, Table 2. Log-rank test showed no statistically significant difference between the two groups (*p* = 0.771). The hazard ratio for GhGI (1.18) was higher than that for ZrGI (0.849), however, this difference was not statistically significant.

**Table 2:** Survival of ZrGI and GhGI restorations

		**ZrGI**	**GhGI**
**Survival time**	Mean (SE)	11.39 (0.31)	11.32 (0.34)
	95% CI	10.78, 11.99	10.65, 11.99
**Log-rank test**	Chi-squared	0.085	
	*p-value*	0.771	
**Hazard Ratio**	HR	0.849	1.18
	95% CI	0.28, 2.56	0.39, 3.55

## Discussion

Management of MIH is a challenge to the pediatric dentist. MIH-affected teeth are more susceptible to caries due to the porous nature of enamel, and PEB that results in denuded dentine [45]. According to the latest guidelines, MIH-affected teeth can be restored with GICs benefiting from their fluoride-releasing and remineralizing potential [5]. However, ongoing research is directed towards combining these qualities with physical properties that enable the restoration to withstand occlusal loading. To the best of our knowledge, this is the only clinical study comparing ZrGI and GhGI restorations in HPFM particularly, in the pediatric population.

Since GICs are moisture-tolerant restorative materials, they do not require strict isolation procedures. Using the ART protocol, the application of both restorative materials in our study was a facile and time-efficient procedure [11]. Multiple dental visits are required to restore MIH-affected teeth using the conventional caries removal method. ART is convenient for managing multiple carious lesions in the same sitting. Saad et al. [46] pointed out that by avoiding additional unnecessary tooth destruction, the ART technique may have a positive effect on the quality of life of affected children. This is very important in dealing with an anxious child with multiple HPFMs especially if pain and sensitivity are involved [47]. The remineralizing potential of GIC has been the focus of several studies [48, 49], making it a suitable restorative material after caries removal using the ART approach in HPFMs. Bonding of restorative materials to hypomineralized enamel is very challenging, since the increased protein content in hypomineralized enamel interferes with bonding strength of adhesives [50]. Micro-mechanical adhesion of resin composite in HPFMs is difficult to achieve since etching of hypomineralized enamel results in fewer resin tags [51]. Some studies advocated the use of 5.25% sodium hypochlorite to improve bonding of composite restorations to HPFMs [52, 53]. ART advocates the use of chemically adhering GIC-based materials, this eliminates the need for dental adhesives. The morphology of HPFMs is unique. Unlike pit and fissure caries, areas of hypomineralized enamel appear sporadically on these molars and do not follow a certain pattern, therefore, following a strict cavity design for the removal of caries and hypomineralized tissue in such cases is not feasible. This is another merit of using the ART approach in HPFMs; only hypomineralized or decomposed tissue is removed, hence the tooth structure is preserved.

There is still insufficient information to support the use of ART restorations in multiple surfaces in permanent posterior teeth [8]. One major limitation of the ART approach is that the size of occlusal access significantly affects the efficacy of demineralized tissue removal [54]. Additionally, caries removal is subjective, and is affected by factors such as illumination of the field, pressure applied by the operator during caries removal, and his/her expertise and training on using the ART approach [55].

In our study, only one HPFM was selected per patient. All patients were given standard oral hygiene instructions at the beginning of the study. Procedures such as preventive GIC-based fissure sealants or fluoride varnishes were not performed to avoid potential confounding effects on the study outcomes, such as sensitivity scores. If any restorative procedures were required in the rest of the molars, they were performed using non-fluoride containing restorative materials. These molars were not included in the study. Evaluating sensitivity in the HPFM was performed by directing the air syringe 1 cm away from the tooth. Therefore, the response obtained was limited to the molar under study to avoid any false-positive results [56].

Frencken and Leal [57] explained that the correct use of the ART approach involves the use of hand instruments only. In their study, Honkala et al. [58] used the ART approach in a clinical setting, unlike other studies where ART was performed in field settings [59, 60]. Burke et al. [61] reported that dentists found that the use of rotary equipment made the procedure quicker and easier. In ART, only unsupported enamel is removed followed by the removal of the affected dentinal tissue exclusively with manual instruments, thus preserving as much structure as possible [57]. In our study, the ART technique was the standard technique utilized for caries removal, however in a few instances (4 patients), the mART [43] was applied. In these cases, the operator realized an alteration in patient’s cooperation and speed was required to finish the restorative procedure. In other instances, the pressure of hand excavation and contact between the metallic instrument and the tooth resulted in a sensitivity response from the patient. Linner et al. [56] investigated hypersensitivity in MIH molars and Steffen et al. [34] reported that hypersensitivity to thermal or mechanical stimuli is a common clinical symptom in MIH.

Regarding the effect of caries removal approach on survival of either restorations, Eden et al. [62] reported that no significant differences in survival rates of Class II composite restorations were found after the use of ART and conventional rotary instruments, and that failures were due to poor bonding of the self-etch adhesive. Louw et al. [63] compared between GIC and compomer restorations, placed using ART and minimally invasive caries removal using rotary instruments. Although success rates were higher using minimally invasive caries removal, there were no significant differences between the two groups, they added that the two approaches were well accepted as complementary for the caries removal process.

In this study, the molars selected according to the MIH-TNI scores 2a, 2b, 4a, and 4b showed hypomineralized enamel and dentin cavitation, mostly on the occlusal surface of the molars. Carious tissue involving hypomineralized enamel was removed with sharp spoon excavators of 1 mm in diameter. The caries removal process followed the principles of Minimum Intervention Dentistry, where the tissue was removed selectively, depending on the cavity depth [42]. If the child complained of pain, local anesthesia was administered. Any residual hypomineralized or unsupported enamel was removed with a hatchet specifically developed for the ART approach. The restoration margin was placed in sound enamel. Fragelli et al. [64] attempted conservative cavities in HPFMs where only carious tissue was removed, however, in their study, residual opacities or hypomineralized enamel was not removed. They concluded that complete removal of the affected enamel should be postponed until the child is mature enough to cooperate. Linner et al. [65] argued that non-invasive restorations in MIH-affected molars result in lower survival rates. In the ART approach, the extent of hypomineralized and carious enamel and dentine dictate the extension of the cavity margins. Since GIC adheres chemically to the tooth structures, no specific alterations in the cavity margins are needed.

The ART evaluation criteria used in this study to evaluate the ZrGI and GhGI restorations is a valid and simplified evaluation tool [66]. It considered not only the condition of the restoration but also the presence of secondary caries.

In this study, we focused on reporting the patients’ perception of pain in restored MIH-affected molars. Our results reveal that caries removal and sealing the HPFM lead to a reduction in pain scores in both groups. Since our results showed no significant difference in pain scores among the groups, therefore, we can claim that ZrGI was as effective as GhGI in reducing pain after removal of carious enamel and dentine and adequately sealed the HPFMs. Both ZrGI and GhGI are chemically setting types of GIC which eliminates variables related to the light-curing process. The significantly higher pain scores in the ZrGI gp. recorded at the 1-week visit could have been due to a number of reasons. There is a probability that higher MIH-TNI scores i.e. a higher degree of hypomineralization was present among the ZrGI group. Afzal et al. [67] concluded that severity of hypomineralization is directly proportional to caries severity and clinical symptoms in MIH-affected teeth. Another reason may be related to the protective coat that was applied to cover the restorations post-operatively, i.e., cocoa butter in the ZrGI gp. as compared to the light-cured resin coat used in the GhGI gp. Post-operative pain, which may persist for up to 1–3 weeks, could be caused by the pressure exerted during hand excavation or any cold stimulus [68, 69]. However, no difference in pain scores was observed between the two groups in subsequent follow-up visits.

The cause of MIH sensitivity is not clear, one hypothesis is that, due to the porosity of enamel, repeated irritating stimuli might cause a subclinical pulp inflammatory response [70]. MIH-associated hypersensitivity results in difficulties in obtaining profound anesthesia which requires the use of adjunct anesthetic techniques [71, 72]. Afzal et al. [67] correlated the severity of MIH with the incidence of hypersensitivity reported in 25.8% of the HPFMs in their study.

The initially high sensitivity scores in both groups were significantly decreased at the 1-week visit in both groups. This might be due to caries excavation process and properly sealing the cavity. This finding is consistent with data published by Fütterer et al. [73], who showed that restorative interventions reduced hypersensitivity and improved the quality of life of MIH-affected children. Interestingly, an increase in sensitivity levels was observed in both groups starting from the 3-month visit and continued throughout the follow-up period. Although this increase was significantly higher in the ZrGI gp. than those in the GhGI gp., sensitivity scores in both groups continued to rise, and no significant difference was found in subsequent follow-up visits. It is worth noting that this coincided with the appearance of white or yellowish-white opacities on the buccal and/or lingual surfaces of the molars, indicating more pronounced manifestations of the hypomineralization process.

The sensitivity reported by the patients during the follow-up visits was in response to the air blast test. When patients were asked about other sensitivity-provoking stimuli, some patients reported sensitivity due to cold drinks and toothbrushing. One patient complained: “ I feel sensitivity in my teeth when I open my mouth to talk”. Another patient reported: “ My teeth were sensitive when I had the flu”, probably due to mouth-breathing. Our findings agree with Raposo et al. [74] who reported that a substantial degree of hypersensitivity persists in restored MIH teeth.

In our study, the increase in hypersensitivity was also associated with evidence of PEB, causing exposure of dentine in scattered areas of the molars. PEB is a sequela of the compromised quality of enamel in HPFMs, which leads to breakdown under occlusal loads [47]. Neves et al. [75] argued this statement and reported that there was no higher chance of PEB in areas exposed to masticatory loads.

Linner et al. [56] reported that teeth with evidence of demarcated opacities were less sensitive than teeth with PEB. It might be argued that the removal of carious dentine and hypomineralized enamel, and placing the restoration helped to overcome pain and sensitivity.

After 12M, the success rates for ZrGI and GhGI restorations were 86.4% and 84.1% respectively. There was no statistically significant difference between both groups, (*p* = 0.765). Zanata et al. [44] reported the 10-year clinical performance of high-viscosity GIC, for single-surface restorations in permanent molars, using the ART approach. The high success rate reported after 12M (98.7%), dropped to (65.2%) after 10 years. Nevertheless, they concluded that the results confirm the potential of the ART approach in restoring and saving posterior permanent teeth. Grossi et al. [10] reported success rates as high as 98% after 12 months using glass hybrid restorations to restore HPFMs following selective caries removal. Durmus et al. [9] after conducting a 24M study, reported that GhGI showed cumulative survival probabilities of 94% at 12 months, and 87.5% after 24 months. Other researchers reported survival rates ranging between 77–88% after 24 months [76, 77]. Sen Yavuz et al. [76] concluded, following their 36-month study, that GhGI placed after selective caries removal was successful in MIH-affected molars. The lower success rates in our study as compared to the studies mentioned above might be due to a difference in sample size, inclusion criteria, study design, or study methodology. In our study, the failed restorations (6 restorations in the ZrGI gp. and 7 in the GhGI gp.) were due to the presence of a marginal defect deeper than 0.5 mm. No restorations in either group were lost or required replacement by another material.

The similar success rates between ZrGI and GhGI in our study imply that ZrGI possesses favorable physical properties. Only one clinical trial, performed by Bayazit et al. [78], attempted to compare ZrGI, glass carbomer, high-viscosity glass ionomer (HVGI), and bulk-fill composite (BC) in first and second permanent molars in adults over a 24M follow-up period. The study reported no significant difference between ZrGI, HVGI, and BC restorations in terms of retention, anatomical form, and marginal discoloration; however, the reported surface texture of HVGI and BC was superior to ZrGI. Several in-vitro studies [79, 80] have investigated properties like flexural and compressive strength, and surface roughness of ZrGI. Chalissery et al. [79] concluded that both compressive and diametral tensile strength of ZrGI were significantly higher than reinforced GIC (Fuji 1X). In their systematic review and network meta-analysis, Manisha et al. [23] concluded that zirconia-reinforced GIC demonstrated more favorable compressive strength values when compared with resin-modified GIC and high-viscosity GIC but less than Compomer and Giomer cements. In another in-vitro study conducted by Kumari and Singh [81], ZrGI yielded better results than conventional GIC (Fuji II), but less than composite (Cention N), in terms of dentine shear bond strength (SBS). Nanavati et al. [82] found no difference in SBS values between ACTIVA™ KIDS BioACTIVE and ZrGI. The nuanced properties of ZrGI may result from the uniform particle size of zirconia particles (ZnO_2_) during the micro-ionization process, which may lead to its clinical durability [23, 83].

In the present study, a coating was used to cover both restorative materials to reduce the initial water absorption to avoid volumetric changes during the setting process of GICs. This could be the cause of the similarity between the number of successful ZrGI and GhGI restorations. Ugurlu et al. [80] reported that applying a coat to ZrGI restorations increased its flexural and compressive strength and decreased its surface roughness after 24 hrs up to 1 year. In their study, surface roughness due to water ageing was seen in uncoated ZrGI restorations.

Our study reveals that the difference in the survival times of ZrGI and GhGI was insignificant. Kemoli et al. [84] stated that marginal gaps reduce a restoration’s survival. The primary causes of marginal deterioration observed in the ZrGI restorations may be related to microleakage, as documented by Patel et al. [85] and Kumari et al. [81], where ZrGI exhibited higher microleakage compared to other types of GIC(Fuji II) and composites (Cention N, and Ceram-x-duo). The reported high microleakage values might be linked to the zirconia filler particles that might affect the chelation reaction, as reported by Baig and Fleming [86]. Contrary to this, Albeshti et al. [83] reported that microleakage values in ZrGI showed no significant differences compared to composite (Filtek™ Z500).

As demonstrated in this study, since there was no difference in pain and sensitivity scores, and clinical performance of both ZrGI and GhGI, therefore, the null hypothesis was not rejected. ZrGI may be considered as a promising restoration in HPFMs. However, neither ZrGI nor GhGI helped to prevent further PEB.

### Limitations

Several studies comparing restorative materials adopted a split-mouth design [76, 87, 88]. In such studies, error variance is reduced, and more powerful statistical tests can be obtained [89]. However, this study was set up to make between-patient comparisons, eliminating the possibility of carry-across effects [90]. The rigorous inclusion criteria and the limited prevalence of MIH challenged the investigator during the recruitment process of this study. The subjective nature of pain/hypersensitivity, and the variability among children caused by age-related anxiety levels also lead to difficulties in reporting the outcomes of this study. Another major limitation was that data concerning the clinical success rate of ZrGI is still scarce in the dental literature, which challenged the verification of our results.

Clinical trials of longer durations and larger sample sizes are recommended to confirm our findings and compare the clinical success of ZrGI in HPFMs with other restorative materials. The longevity of ZrGI in stress-bearing areas should be further investigated. Future clinical trials could highlight the incidence of PEB and hypersensitivity, two very characteristic phenomena associated with MIH. We also recommended investigating the use of ZrGI as a fissure sealant to compare its retention rate with other glass ionomer-based or resin-based fissure sealants in HPFMs.

## Conclusions

Based on the results of this randomized clinical trial, the following conclusions can be made:

1. Zirconia-reinforced glass ionomer and glass-hybrid glass ionomer performed the same clinically, regarding the ability to reduce perception of pain and sensitivity, in HPFMs.
2. Both restorations showed satisfactory success rates but did not prevent further enamel breakdown on other aspects of MIH-affected molars.

## Supplementary Information

Below is the link to the electronic supplementary material.

Supplementary file1 (PDF 256 KB)

## Data Availability

No datasets were generated or analysed during the current study.
