# Supplementary material for: Zirconia-reinforced glass ionomer restorations in molar incisor hypomineralization: a randomized controlled clinical trial
Source: Clin Oral Investig. 2025 May 8;29(6):290. doi: 10.1007/s00784-025-06352-y (PMC12058950; doi:10.1007/s00784-025-06352-y)
Supplement: Supplementary file 1 — Supplementary file1 (PDF 256 KB) [file 784_2025_6352_MOESM1_ESM.pdf]

Supplementary Information

Clinical Oral Investigations

Zirconia-reinforced glass ionomer restorations in molar incisor hypomineralization: a randomized controlled clinical trial

Reham A. Mahfouz[1], Azza G. Hanno[2], Amina M. Abd El Rahman[2]

[1] PhD candidate, Department of Pediatric Dentistry, Faculty of Dentistry, Alexandria University, El Khartoum Square, Alexandria 21131, Egypt
[2] Professor, Department of Pediatric Dentistry, Faculty of Dentistry, Alexandria University, El Khartoum Square, Alexandria 21131, Egypt

Corresponding author: Reham A. Mahfouz
reham.mahfouz@alexu.edu.eg
Orchid iD: 0009-0004-2506-671

Table S1- MIH Treatment Need Index[1]

Index	Definition
Index 0	No MIH, clinically free of MIH
Index 1	MIH without hypersensitivity, without defect
Index 2	MIH without hypersensitivity, with defect
2a	<1/3 defect extension
2b	>1/3 < 2/3 defect extension
2c	>2/3 defect extension or/and defect close to the pulp or extraction or atypical restoration
Index 3	MIH with hypersensitivity, without defect
Index 4	MIH with hypersensitivity, with defect
4a	<1/3 defect extension
4b	>1/3 < 2/3 defect extension
4c	>2/3 defect extension or/and defect close to the pulp or extraction or atypical restoration

Table S2 ART Evaluation Criteria [2]

Code	Criterion	Definition
0	Present, satisfactory	Successful
1	Present, slight defect at the cavity margin of less than 0.5 mm; no repair is needed	Successful
2	Present, marginal defect deeper than 0.5 mm	Failed
3	Partially present, restoration and/or tooth breakdown	Failed
4	Not present, restoration missing	Failed
5	Not present, other restorative treatment has been performed	Failed
6	Not present, tooth has been extracted	Failed
7	Pulpal involvement	Failed
C	Caries present	Failed

Table S3 Demographic data of the study groups

**Variables**		**ZrGI (Test) (n=44)**	**GhGI (Control) (n=44)**	***p-value***
Age in years	Mean ±SD	7.88 ±0.73	7.85 ±1.00	0.904
Gender: n (%)	Males	16 (36.4%)	24 (54.5%)	0.087
	Females	28 (63.6%)	20 (45.5%)	
Arch: n (%)	Lower	24 (54.5%)	24 (54.5%)	1.00
	Upper	20 (45.5%)	20 (45.5%)	

Table S4 Pairwise comparisons showing pain scores within the ZrGI and GhGI groups

**Groups**	**Compared to**	***p-value***	
		**ZrGI**	**GhGI**
Baseline	1 Week	<0.0001*	<0.0001*
	3 Months	<0.0001*	<0.0001*
	6 Months	<0.0001*	<0.0001*
	9 Months	<0.0001*	<0.0001*
	12 Months	<0.0001*	0.001*
1 Week	3 Months	1.00	1.00
	6 Months	1.00	1.00
	9 Months	1.00	1.00
	12 Months	1.00	1.00
3 Months	6 Months	1.00	1.00
	9 Months	1.00	1.00
	12 Months	1.00	1.00
6 Months	9 Months	1.00	1.00
	12 Months	1.00	1.00
9 Months	12 Months	1.00	1.00

*Statistically significant difference at *p*-value<0.05

Table S5 Pairwise comparisons showing sensitivity scores within the ZrGI and GhGI groups

**Groups**	**Compared to**	***p-value***	
		**ZrGI**	**ZrGI**
Baseline	1 Week	<0.0001*	<0.0001*
	3 Months	<0.0001*	<0.0001*
	6 Months	0.008*	<0.0001*
	9 Months	0.366	0.001*
	12 Months	0.604	0.026*
1 Week	3 Months	1.00	1.00
	6 Months	1.00	1.00
	9 Months	0.844	1.00
	12 Months	0.525	0.489
3 Months	6 Months	1.00	1.00
	9 Months	0.251	1.00
	12 Months	0.143	0.604
6 Months	9 Months	1.00	1.00
	12 Months	1.00	1.00
9 Months	12 Months	1.00	1.00

*Statistically significant difference at *p*-value<0.05

S1.1 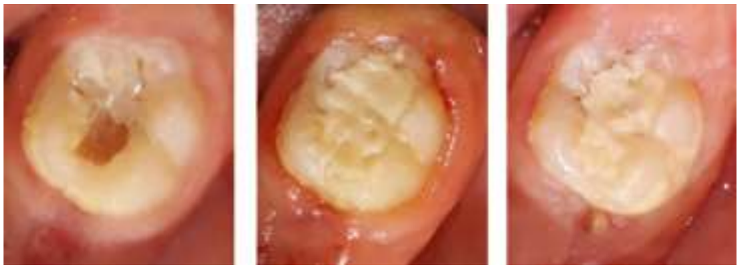

S1.2 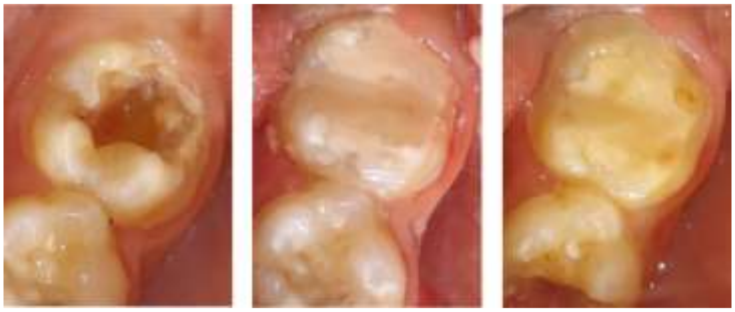

Figures S1.1 and S1.2 Hypomineralized permanent first molars restored with ZrGI preoperative, postoperative, and after 12 months

S2.1 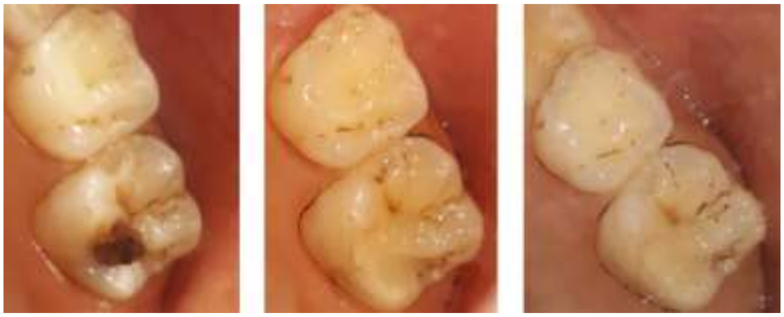

S2.2 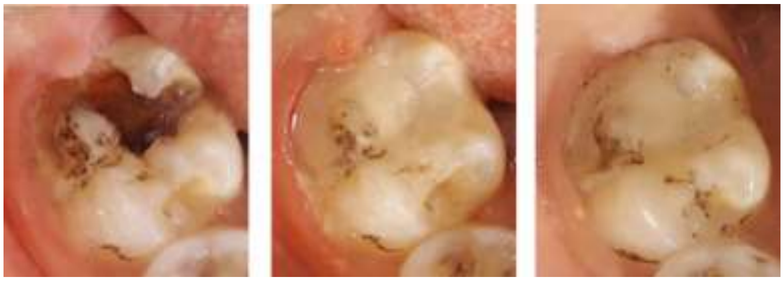

Figures S2.1 and S2.2 Hypomineralized permanent first molars restored with GhGI preoperative, postoperative and after 12 months

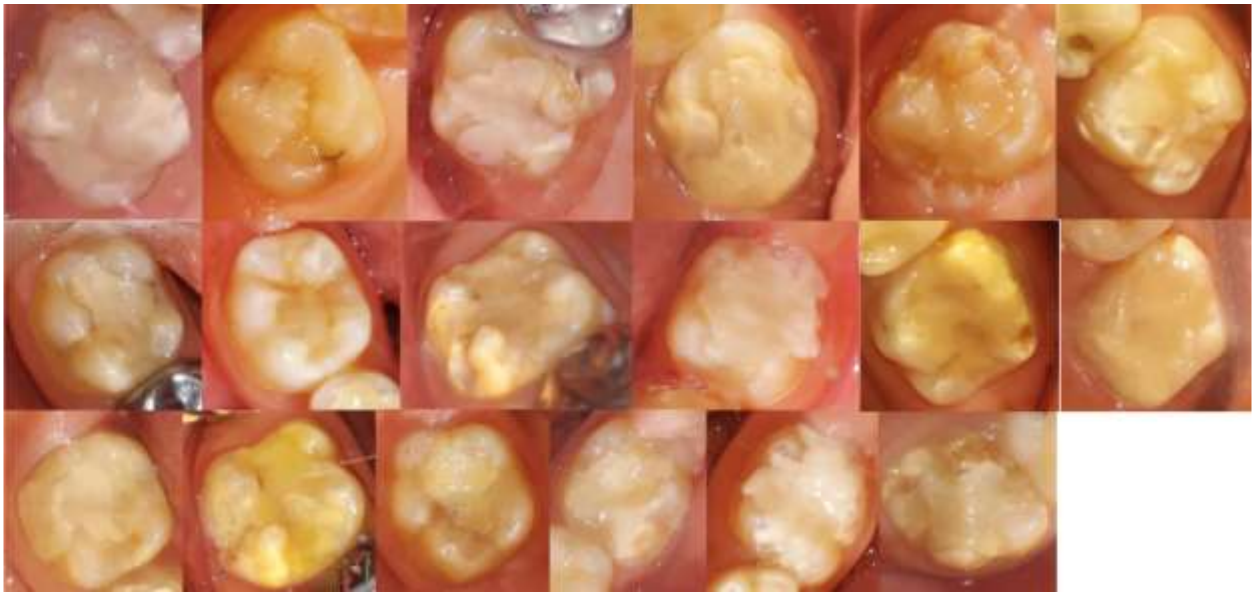

Figure S3 Hypomineralized molars restored with ZrGI after the 12M follow-up period

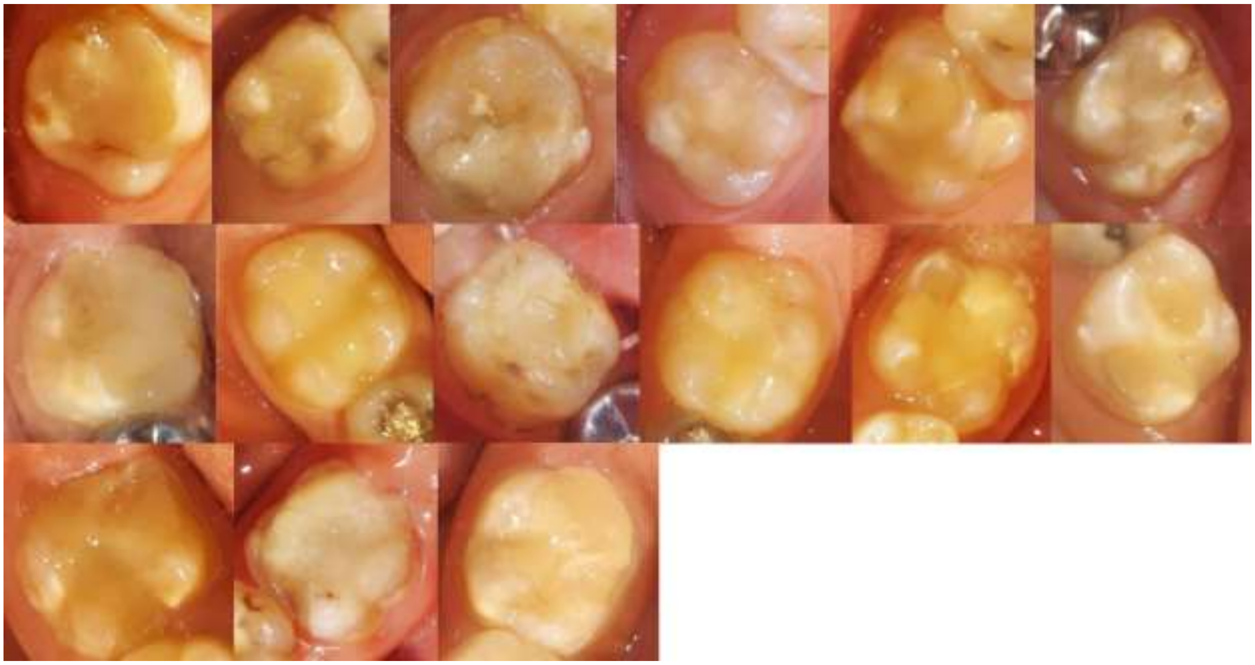

Figure S4 Hypomineralized molars restored with GhGI after the 12M follow-up period

Table S6 ART success/failure rates in the ZrGI and GhGI groups

**Time Intervals**	**ZrGI (Test) (n=44)**		**GhGI (Control) (n=44)**		***p-value***
	**Success n (%)**	**Failure n (%)**	**Success n (%)**	**Failure n (%)**	
3 Months	43 (97.7%)	1 (2.3%)	42 (95.5%)	2 (4.5%)	1.00‡
6 Months	41 (93.2%)	3 (6.8%)	41 (93.2%)	3 (6.8%)	1.00‡
9 Months	39 (88.6%)	5 (11.4%)	39 (88.6%)	5 (11.4%)	1.00‡
12 Months	38 (86.4%)	6 (13.6%)	37 (84.1%)	7 (15.9%)	0.765¥
***p*-value**	0.015*		0.015*		

‡Fisher's Exact test, ¥Pearson Chi Square test

Table S7 Pairwise comparisons regarding ART scores within ZrGI and GhGI groups

**Groups**	**Compared to**	***p-value***	
		**ZrGI**	**GhGI**
3 Months	6 Months	1.00	1.00
	9 Months	0.105	0.448
	12 Months	0.018*	0.018*
6 Months	9 Months	1.00	1.00
	12 Months	0.448	0.105
9 Months	12 Months	1.00	1.00

*Statistically significant difference at p-value<0.05

REFERENCES

1. Steffen R, Krämer N,Bekes K (2017) The Würzburg MIH concept: the MIH treatment need index (MIH TNI) A new index to assess and plan treatment in patients with molar incisior hypomineralisation (MIH). European Archives of Paediatric Dentistry 18:355-361
2. Zanata RL, Fagundes TC, Freitas MC, Lauris JR,Navarro MF (2011) Ten-year survival of ART restorations in permanent posterior teeth. Clin Oral Investig 15:265-71
